# Reviewed article: Biase, TMMA; Silva, MT; Galvão, TF. Selective reporting of adverse renal reactions associated with selective cyclooxygenase-2 inhibitors in randomized clinical trials: a cross-sectional study, 2023. Epidemiol Serv Saude. 2026:35;e20260191

**DOI:** 10.1590/S2237-96222026v35e20260191.a

**Published:** 2026-11-09

**Authors:** Vanessa Gomes Lima

**Affiliations:** 1 Universidade Estadual de Campinas, Campinas, SP, Brazil Universidade Estadual de Campinas Campinas SP Brazil

## Peer review A

### Questionnaire

Does the manuscript contain new and significant information to justify publication? Yes 

Does the Abstract (Summary) clearly and accurately describe the content of the article? No

Is the problem significant and concisely stated? Yes 

Are the methods described comprehensively? No

Are the interpretations and conclusions justified by the results? Yes 

Is adequate reference made to other work in the field? Yes 

### Manuscript Structure:

Length of article is: Adequate

Number of tables is: Adequate

Number of figures is: Adequate

### Rating

Interest Good

Quality Good

Originality Good

Overall Good

Recommendation: **Major Revision**

## Comments

Embora o objetivo geral esteja compreensível, a formulação da pergunta de pesquisa ainda pode ser refinada. Uma sugestão é falar de modo mais direto que a unidade analítica do estudo é cada ensaio clínico/publicação incluído na revisão sistemática, e não os participantes dos ensaios. Também recomendo detalhar melhor a definição operacional do desfecho principal (“relato seletivo”), incluindo os critérios de classificação e sua aplicação.

Outro ponto interessante é dizer que o estudo teve dois objetivos distintos, estimar a frequência do fenômeno e explorar fatores associados, ou se a análise de associações foi estritamente exploratória. Esse ajuste beneficiaria especialmente o resumo e os parágrafos finais da introdução.

A estratégia analítica baseada apenas em teste t e qui-quadrado é insuficiente para sustentar interpretações mais robustas sobre fatores associados.

Seria bom incluir medidas de efeito com IC95%, avaliar a adequação dos testes em face de células pequenas e, empregar modelo multivariável ou análise de sensibilidade que reduza o risco de confusão residual.

Seria importante incluir medidas de efeito e IC95% nas tabelas analíticas, em vez de apresentar apenas p-valores.

Os resultados sustentam a afirmação de que o relato seletivo foi frequente, mas não permitem afirmar, com a formulação atual, que houve influência de políticas editoriais e patrocínio. Recomendo substituir linguagem causal ou insinuativa por formulações mais cautelosas, compatíveis com análise observacional exploratória.

Descrever com mais precisão como o desfecho foi classificado e revisar a conclusão para evitar extrapolação causal. O resumo já traz números importantes, mas os métodos ainda podem ser descritos com maior precisão.

O tema é relevante e o foco em relato seletivo de reações adversas renais em ensaios com coxibs tem potencial de originalidade. No entanto, essa contribuição precisa ser melhor destacada na introdução e na discussão, com explicitação mais clara do que este estudo acrescenta à literatura sobre viés de relato, especialmente em comparação com investigações que confrontam protocolos e artigos publicados.

Discutir a validade externa com mais cuidado. O estudo tem interesse além de um contexto local, mas o corpus é restrito a ensaios com coxibs e eventos renais, o que limita generalizações amplas para toda a comunicação científica ou para outras classes terapêuticas. Recomendo explicitar melhor o alcance e os limites da extrapolação.

Os resultados são relevantes, mas ainda não estão plenamente desenvolvidos. O manuscrito permanece excessivamente apoiado em análise descritiva e bivariada. Para fortalecer o caráter conclusivo, sugiro ampliar a interpretação analítica, incluir medidas de efeito, explorar análises de sensibilidade e discutir de forma mais sistemática a robustez dos achados.

A descrição metodológica precisa ser expandida. É essencial detalhar a revisão sistemática de origem, o processo de derivação das variáveis, os critérios exatos de classificação do relato seletivo e a forma de obtenção das variáveis editoriais e bibliométricas. Também seria recomendável alinhar o texto a um guia de relato apropriado para estudos observacionais.

No formato atual, não está suficientemente claro como se reduziu o risco de erro de classificação do desfecho, nem como foi enfrentada a possibilidade de confusão entre características dos estudos. Recomendo uma seção mais desenvolvida sobre validade interna.

Recomendo justificar melhor a suficiência do corpus de 49 ensaios para as análises propostas, reconhecendo o poder estatístico limitado para múltiplas comparações e eventual ajuste multivariável. Também convém discutir que o conjunto de estudos representa os ensaios incluídos na revisão sistemática, e não necessariamente todo o universo de evidência sobre o tema.

Recomendo reforçar as implicações para transparência em segurança de medicamentos e avaliação crítica de evidências para tomada de decisão, sem extrapolar além do que os resultados permitem. A conclusão deve responder diretamente ao objetivo, com redação mais prudente.

Evitar expressões que insinuem causalidade sem suporte analítico correspondente.

Há necessidade de revisão gramatical e editorial. Por exemplo, a expressão “O relato seletivo reações renais” precisa correção. Sugiro uma revisão final cuidadosa de ortografia, concordância, padronização de p-valores e uniformidade terminológica. Há também problemas de clareza e escolha vocabular. Por exemplo, “A omissão foi mais frequente em periódicos com menor fator de impacto” é compreensível, mas “omissão” aparece sem ancoragem imediata e poderia ser retomada de modo mais explícito, como “o relato seletivo foi mais frequente... o espaçamento de valores numéricos, como “190,7±155, 3”, “155,1±91, 4” e “n =11”, que precisam de uniformização

